# Five new species of the genus Cryptopimpla Taschenberg (Hymenoptera, Ichneumonidae) with a key to species known from China

**DOI:** 10.3897/zookeys.117.1302

**Published:** 2011-06-08

**Authors:** Mao-Ling Sheng

**Affiliations:** General Station of Forest Pest Management, State Forestry Administration, Shenyang, Liaoning, 110034, China

**Keywords:** Banchinae, taxonomy, parasitoid wasp, identification

## Abstract

Eight species of the genus *Cryptopimpla* Taschenberg, 1863 are reported from China, five of them new to science: *Cryptopimpla flavipedalis* Sheng, **sp. n.**, collected from Ningxia Hui Autonomous Region, and *Cryptopimpla rufipedalis* Sheng, **sp. n.** collected from Jilin Province, both from the Palaearctic part of China. *Cryptopimpla. carinifacialis* Sheng, **sp. n.**, *Cryptopimpla flavifacialis* Sheng, **sp. n.**and *Cryptopimpla maculifacialis* Sheng, **sp. n.** were collected from Jiangxi Province in the Oriental part of China. A key to the species of *Cryptopimpla* known from China is provided.

## Introduction

According to recent publications (Kuslitzky 2007, Sheng et al. 2005, 2009, Yu et al. 2005), the genus *Cryptopimpla* Taschenberg, 1863, belonging to the tribe Atrophini of the subfamily Banchinae (Hymenoptera, Ichneumonidae), comprises 41 species, of which 13 are from the Oriental Region, 20 from the Palaearctic, five from the Nearctic, one is Holarctic, one is Nearctic and Neotropical, and one is from the Ethiopian region. Four species of *Cryptopimpla* have been reported from China (Sheng and Zheng 2005, Sheng and Sun 2009). The status of the genus was elucidated by Townes (1970) and by Chandra and Gupta (1977).

The Oriental species of *Cryptopimpla*Taschenberg were described or redescribed and keyed by Chandra and Gupta (1977). Kuslitzky (2007) provided a key to the banchine genera and species of the Russian Far East. Two species have been described from Japan (Momoi 1970). The European species were catalogued by Aubert (1978). So far, species reported from China were from Henan, Inner Mongolia, Jilin and Taiwan. China is very large and spans two Regions, the Oriental and Palaearctic regions. The genus has not been studied thoroughly in either the Oriental or Palaearctic regions of China.

In the last four years the author has been exploring Jiangxi Province, situated in the northern border of the Oriental part of China, Jilin Province and Ningxia Hui Autonomous Region, both situated at the southern border of the Palaearctic part of China, and has collected large numbers of ichneumonids. New discoveries have been reported (Sheng et al. 2009, 2010, 2011), and will be reported successively. In this article, five new species of *Cryptopimpla* are reported, of which one, collected from Jilin Province in the Palaearctic part of China, was previously mistaken as *Cryptopimpla helvetica* Brauns.

## Materials and methods

Specimens were collected using entomological nets in the forests of Quannan, Ji’an and Qianshan Counties, Jiangxi Province; Liupanshan Natural Reserve, Ningxia Hui Autonomous Region; Baihe, Jilin Province (CHINA).

Images of whole bodies were taken using a CANON Power Shot A650 IS. Other images were taken using a Cool SNAP 3CCD attached to a Zeiss Discovery V8 Stereomicroscope and captured with QCapture Pro version 5.1.

The morphological terminology is mostly that of Gauld (1991). Wing vein nomenclature is based on Ross (1936) and the terminology on (Mason (1986, 1990).

Type specimens are deposited in the Insect Museum, General Station of Forest Pest Management, State Forestry Administration, People’s Republic of China.

### Cryptopimpla

Taschenberg, 1863

http://species-id.net/wiki/Cryptopimpla

Cryptopimpla Taschenberg, 1863. Zeitschrift für die Gesammten Naturwissenschaften, 21:292. Type-species: *Phytodietus blandus* Gravenhorst.

#### Diagnosis.

Upper tooth of mandible longer than lower tooth. Genal carina joining hypostomal carina above base of mandible. Occipital carina complete. Without epomia. Lower half of mesopleuron weakly convex. Posterior transverse carina of propodeum usually present. Propodeal spiracle round or slightly elongate. Areolet present, front side truncate or pointed. 2m-cu with two closely spaced bullae or with one that is 0.5 to 1.0 times as long as the section of 2m-cu behind bulla. First tergum evenly and strongly tapered toward base. Apical portion of metasoma weakly to strongly compressed. Ovipositor sheath approximately 0.6 times as long as hind tibia. Subapical portion of ovipositor with a dorsal notch.

#### Key to species of the genus known from *Cryptopimpla* China

**Table:** 

1	Claws pectinate.	2
–	Claws simple.	5
2	Areolet pentagonal, with 3-Rs distinctly present. Mesosoma, metasoma, hind coxa and femur entirely black.	*Cryptopimpla henanensis* Sheng
–	Areolet quadrilateral, 3-Rs lacking, or areolet very small and obliguely elongate. Body, at least mesosoma or metasoma, with yellow or white spots, or hind coxa and femur light-colour.	3
3	Areolet rather small, obliquely elongate. First tergum 2.5 times as long as apical width	*Cryptopimpla taiwanensis* (Momoi)
–	Areolet relatively big, not so obliquely elongate. First tergum usually short, not more than 2.0 times as long as apical width, or areolet petiolate	4
4	Face with strong median carina (Fig. 2). Areolet petiolate. Face black with latero-median yellowish white spots. Median portion of flagellomeres white. Front and middle femora reddish brown, hind femur brownish black (Fig. 1). Basal and apical portions of tergum 1, apical portions of terga 2, 3, 7 and 8 white.	*Cryptopimpla carinifacialis* Sheng, sp. n.
–	Face slight convex, without median carina (Fig. 12). Areolet sessile. Face yellowish white, upper margin irregularly black. Flagellomeres without white band. All femora yellowish white (Fig. 11). Terga entirely black.	*Cryptopimpla flavipedalis* Sheng, sp. n.
5	Face, mesosoma and all terga entirely black.	*Cryptopimpla brevis* Sheng
–	Face yellow, or black with a light-coloured spot. Mesosoma with yellow spots. Median terga reddish brown, or apical margins of terga white or whitish yellow	6
6	Face black (Fig. 21), median sections of inner orbits more or less yellow. Median terga reddish brown (Fig. 20).	*Cryptopimpla rufipedalis* Sheng, sp. n.
–	Face yellow, or yellowish white with median longitudinal black band. Terga black, hind margins widely yellowish white.	7
7	First tergum 1.6 times as long as apical width. Face white with median longitudinal black band (Fig. 17). Flagellomeres 10 to 16 white. Scutellum yellowish white.	*Cryptopimpla maculifacialis* Sheng, sp. n.
–	First tergum 1.8 to 1.9 times as long as apical width. Face yellow (Fig. 7). Median to subapical portion of flagellomeres yellowish brown. Scutellum black.	*Cryptopimpla flavifacialis* Sheng, sp. n.

### Cryptopimpla carinifacialis

Sheng, sp. n.

urn:lsid:zoobank.org:act:A953FD9F-994C-4541-8B5F-8E0A4F57037C

http://species-id.net/wiki/Cryptopimpla_carinifacialis

Figs 1–5

#### Etymology.

The name of the new species is based on the median longitudinal carina of the face.

#### Material examined.

*Holotype*: female, CHINA: Wuyishan Natural Reserve, 1200m, Qianshan County, Jiangxi Province, 11 July 2009, leg. Zhi-Ping Zhong.

#### Diagnosis.

Median portion of face strongly convex, with distinct median longitudinal carina. Areolet petiolate. Scutellum and postscutellum with dense punctures. Claws pectinate. Hind wing vein 1/cu approximately 3.8 times as long as cu-a. First tergum approximately 2.5 times as long as apical width, slightly narrowed towards base. Ovipositor almost straight. Face black with latero-median yellowish white spots.

#### Description.

Female. Body length about 10.0 mm. Fore wing length about 8.0 mm. Ovipositor sheath length about 2.0 mm.

**Head.** Inner margins of eyes parallel. Face and clypeus with fine leathery texture. Face (Fig. 2) approximately 1.38 times as wide as long, median portion strongly convex longitudinally, with distinct median longitudinal carina; upper-lateral portion longitudinally concave; medially with dense punctures, distance between punctures 0.1 to 0.2 times diameter of puncture; laterally with relatively sparse punctures, distance between punctures 0.5 to 1.0 times diameter of puncture. Clypeus approximately 2.2 times as wide as long, almost smooth, strongly convex, basal portion with sparse punctures, apical median portion slightly concave; apical margin thick and convex, with brown hairs. Mandible strong, subapical portion with sparse and shallow punctures. Teeth sharp, upper tooth distinctly longer than lower tooth. Malar space, gena, vertex and frons with fine leathery texture. Malar space approximately 0.55 times as long as basal width of mandible, with indistinct and fine punctures. Gena directly convergent posteriorly, with fine punctures, distance between punctures 0.2 to 2.0 times diameter of puncture, but gradually more densely punctate towards lower portion. Vertex (Fig. 3) with punctures slightly larger than that of gena, distance between punctures 0.5 to 2.5 times diameter of puncture. Interocellar area weakly convex. Postocellar line approximately as long as ocular-ocellar line. Frons almost flat, sublateral portion with dense and unclear punctures, distance between punctures 0.1 to 0.5 times diameter of puncture, almost contacting each other transversely. Lower-median portion with longitudinal wrinkles. Antenna with 47 flagellomeres, each segment longer than wide. Inner profile of basal half of flagella with a distinctive structure, a strong longitudinal carina (Fig. 3a). Ratio of length from first to fifth flagellomeres: 8.8:6.3:5.7:5.3:5.0. Occipital carina complete, lower end joining hypostomal carina slightly above base of mandible.

**Mesosoma.** Anterior margin of pronotum almost smooth, with indistinct punctures. Laterally concave with short transverse wrinkles. Upper-posterior portion with even and dense punctures, distance between punctures approximately 0.2 times diameter of puncture. Mesoscutum evenly convex, rough, with punctures denser than on upper-posterior portion of pronotum. Without notaulus. Scutellum evenly convex, with punctures as mesoscutum. Postscutellum more convex, with dense and large punctures, larger than on scutellum, latero-anterior portion concave. Mesopleuron (Fig. 4) with dense punctures, distance between punctures 0.1 to 0.6 times diameter of puncture. Upper end of epicnemial carina reaching about lower 0.2 level of front margin of mesopleuron, distant from front margin of mesopleuron. Mesopleural fovea round, deep. Without speculum. Metapleuron with denser and finer punctures than mesopleuron. Submetapleural carina complete, triangularly convex anteriorly. Without juxtacoxal carina. Wings brownish hyaline. Vein 1cu-a distal of 1/M, distance between them approximately 0.26 times length of 1cu-a. Areolet quadrate, petiolate, vein 3rs-m distinctly longer than 2rs-m, receiving vein 2m-cu approximately 0.8 distance from vein 2rs-m to 3rs-m. Vein 2-Cu slightly longer than 2cu-a. Hind wing vein 1/cu about 3.8 times as long as cu-a. Legs comparatively long. Ratio of length of hind tarsomeres 10.0:4.3:3.1:1.5:2.3. Claws densely pectinate. Propodeum (Fig. 5) evenly convex, rough, with dense and indistinct punctures. Median point and lateral portion at the place of apophysis of posterior transverse carina and apical section of pleural carina present. Propodeal spiracle elongate, approximately 2 times as long as maximum width.

**Metasoma.** First tergum approximately 2.5 times as long as apical width, slightly narrowed towards base, with distinct and dense punctures. Without median dorsal carina. Basal portion of dorsolateral carina, basal of spiracle, present. Spiracle very small, round, evidently before middle of tergum. Second tergum about 1.1 times as long as apical width, with dense punctures, distance between punctures 0.1 to 0.3 times diameter of puncture, but gradually finer and more sparsely punctate towards apical margin, basal margin with semicircular thyridium. Third tergum with finer punctures than second tergum, lateral and apical portion weakly, sparsely punctate. Fourth tergum slightly rough, indistinctly punctate. Following terga smooth, with more or less clear transverse lines. Ovipositor sheath approximately 0.55 times as long as hind tibia, approximately as long as first tergum. Ovipositor strongly compressed, almost straight.

**Figures 1–5.**
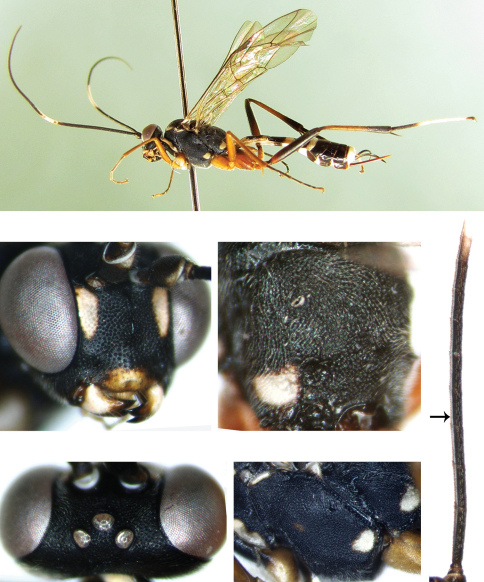
*Cryptopimpla carinifacialis* sp. n. Holotype. Female **1** Body, lateral view **2** Face **3** Vertex **3a** Basal portion of flagellum **4** Mesopleuron **5** Propodeum.

**Color.** (Fig. 1). Black, except the following. White or yellowish white portions: ventral-apical portions of scape and pedicel, apical portion of flagellomere 9, flagellomeres 10 to 16, basal portion of 17, latero-median spots of face, main portion of clypeus, mandible except teeth, maxillary palpus, labial palpus except apical segment grayish brown, front margin and upper-posterior corner of pronotum, elongate spots on latero-anterior portion of mesoscutum, subalar ridge, scutellum except anterior-median portion, spots of posterior portions of mesopleuron and metapleuron, apical part of basitarsus 1, and 2 to 4 entirely, basal, lateral and apical portions of first tergum, posterior margins of terga 2 and 3 widely, 7 and 8 mainly. A small spot near front median portion of mesopleuron vaguely reddish brown. Front and middle coxae and trochanters, ventral profiles of hind coxae, yellowish brown. Front and middle femora, hind coxae, trochanters and base of femora reddish brown. Front and middle tibiae and basitarsus, about basal 0.7 of hind tibiae darkish brown. Stigma blackish brown. Veins brownish black.

#### Remarks.

This new species can be easily distinguished from other species of this genus as the face has a distinct median longitudinal carina, the areolet is clearly petiolate, the first tergum is slightly narrowed towards the base and the inner profile of the basal half of the flagella has a particular structure, a strong longitudinal carina.

### Cryptopimpla flavifacialis

Sheng, sp. n.

urn:lsid:zoobank.org:act:83E14931-95D2-4DF9-80F4-EE2DBBA88EF8

http://species-id.net/wiki/Cryptopimpla_flavifacialis

Figs 6–10

#### Etymology.

The specific name is derived from the face being fulvous.

#### Material examined.

*Holotype*: female, CHINA: Sanjiaotan, 335m, Quannan, Jiangxi Province, 24 March 2009, leg. Shi-Chang Li. *Paratypes*: 1 female, CHINA: Shuangjiang Forest Farm, 174m, Ji’an, Jiangxi Province, 9 April 2009, leg. Da-Lin Li; 1 female, CHINA: Laomaotu, 340m, Quannan, Jiangxi Province, 7 April 2009, leg. Shi-Chang Li.

#### Diagnosis.

Face whitish yellow. Hind coxae brownish red entirely. Basal portion of lower margin of mandible with distinct semitransparent edge. Antenna with 33 flagellomeres. Mesosoma shining. Claws simple. Ovipositor strongly upcurved.

#### Description.

Female. Body length 8.5 to 9.5 mm. Fore wing length 6.5 to 7.5 mm. Ovipositor sheath length 1.5 to 2.0 mm.

**Head.** Face (Fig. 7) approximately 1.4 times as wide as long, with even, dense and fine punctures, distance between punctures 0.2 to 0.5 times diameter of puncture, median portion slightly convex, upper margin medially concave, with small tubercle. Clypeus smooth, evenly convex, basal portion with sparse, fine punctures, apical portion almost impunctate; apical margin thick, convex, with relatively long hairs. Mandible strong, with weak, fine punctures, distance between punctures 0.5 to 1.0 times diameter of puncture. Basal portion of lower margin with semitransparent edge. Upper tooth slightly longer than lower tooth. Malar space 0.47 to 0.48 times as long as basal width of mandible, slightly coarse, with shallow, indistinct punctures. Gena with fine leathery texture and uneven, fine punctures, distance between punctures 0.2 to 2.5 times diameter of puncture. Vertex (Fig. 8) with texture as that of gena, interocellar area with dense punctures, distance between punctures less 0.5 times diameter of puncture; Postocellar line 0.9 to 1.0 times as long as ocular-ocellar line. Frons almost flat, with relatively deep, dense punctures, lower portion near antennal socket smooth, impunctate. Antenna with 33 flagellomeres, each segment longer than wide; ratio of length from first to fifth flagellomeres: 2.0:1.8:1.7:1.6:1.5. Occipital carina complete, lower end joining hypostomal carina slightly above base of mandible.

**Mesosoma.** Mesosoma smooth, with dense punctures, distance between punctures less than diameter of puncture. Anterior margin of pronotum with elongate punctures; laterally concave with short longitudinal wrinkles. Notaulus unclear. Scutellum evidently convex, without lateral carina. Postscutellum transverse. Median portion of mesopleuron (Fig. 9) slightly convex. Upper end of epicnemial carina reaching level of lower third of hind margin of pronotum, comparatively far distant from front margin of mesopleuron. Without speculum. Metapleuron with punctures as mesopleuron. Front portion of submetapleural carina anteriorly convex. Wings brownish hyaline. Vein 1cu-a distal of 1/M, distance between them approximately 0.4 times length of 1cu-a. Areolet quadrate, vein 3rs-m distinctly longer than 2rs-m, receiving vein 2m-cu at 0.6 to 0.7 distance from vein 2rs-m to 3rs-m. Vein 2-Cu slightly longer than 2cu-a. Hind wing vein 1/cu about 2 times as long as cu-a. Legs comparatively robust. Ratio of length of hind tarsomeres 6.2:3.8:1.9:1.0:1.4. Claws simple. Propodeum (Fig. 10) evenly convex, with punctures as on mesosoma. Pleural carina and median portion of posterior transverse carina present. Propodeal spiracle small, oval.

**Metasoma.** First to third terga with dense punctures, but slightly sparser than on mesosoma. First tergum 1.8 to 1.9 times as long as apical width, evenly narrowed towards base; median longitudinal portion nearly impunctate; lateral portion behind spiracle with fine longitudinal wrinkles. Spiracle convex, slightly before middle of tergum. Second tergum about as long as apical width. Thyridium almost semicircular. Basal width of third tergum slightly wider than apical width, 0.9 times as long as widest portion, latero-basally concave. Fourth tergum with very weak and fine punctures. Following terga indistinctly punctate. Ovipositor sheath approximately 0.7 to 0.8 times as long as hind tibia, 1.2 to 1.3 times as long as first tergum. Ovipositor strongly upcurved and compressed.

**Figures 6–10.**
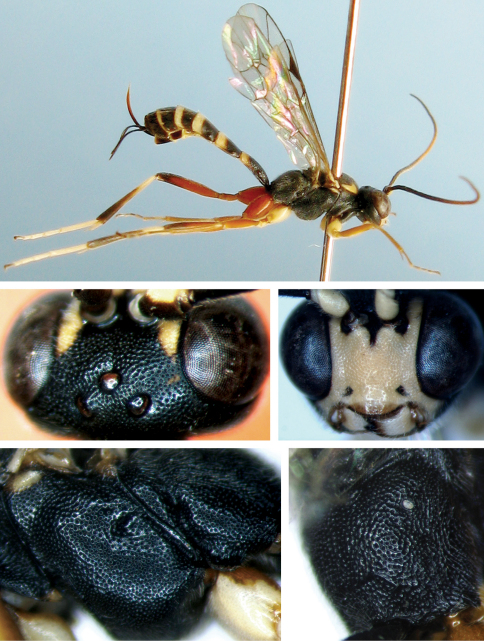
*Cryptopimpla flavifacialis* sp. n. Female **6** Body, lateral view **7** Face **8** Vertex **9** Mesopleuron **10** Propodeum.

**Color.** (Fig. 6). Black, except the following. Median to subapical portion of flagellum yellowish brown. Ventral profile of scape and pedicel, face except upper-median margin with longitudinal dark groove and a small tubercle, malar space, frontal orbit, latero-anterior margin of mesoscutum, tegula, subalar ridge, ventral profiles and apices of front and middle coxae, ventral profiles of front and middle trochanters and femora, posterior margin of each tergum whitish yellow. Maxillary and labial palpi bright yellow with brown flecks. Legs reddish brown, basal portions of front and middle tibiae, apices of first to fourth front and middle tarsomeres, basal portion of hind tibia, first to fourth hind tarsomeres white. Hind coxa brownish red. Dorsal profile of hind trochanter, apex of hind femur, apical half of hind tibia and fifth tarsomere black to brownish black. Stigma and veins brownish black.

#### Variation.

The specimen from Shuangjiang Forest Farm, Ji’an, Jiangxi Province, has the first tergum more or less and irregularly darkish red.

#### Remarks.

Similar to *Cryptopimpla taiwanensis* (Momoi, 1968), but can be distinguished from the latter by the antenna with 33 flagellomeres, simple tarsal claws, first tergum 1.8 to 1.9 times as long as apical width, second tergum about as long as apical width, ovipositor strongly upcurved, first tergum except apical portion and terga 6 to 8 black, except hind margin narrowly whitish yellow. *Cryptopimpla taiwanensis*: antenna with 48 flagellomeres, tarsal claws strongly pectinate, first tergum about 2.5 times as long as apical width, second tergum about 1.3 times as long as apical width, ovipositor straight, basal portion of first tergum and terga 6 to 8 entirely, yellow.

### Cryptopimpla flavipedalis

Sheng sp. n.

urn:lsid:zoobank.org:act:63B65E54-E7EB-481B-8B2D-03A3EAF9A1CB

http://species-id.net/wiki/Cryptopimpla_flavipedalis

Figs 11–15

#### Etymology.

The specific name is derived from the entirely yellow legs.

#### Material examined.

*Holotype*: female, CHINA: Liupanshan, 1280m, Ningxia Hui Autonomous Region, 25 August 2005, leg. Mao-Ling Sheng.

#### Diagnosis.

Gena with fine granular texture, almost impunctate. Mesosoma with fine leathery texture. Legs yellowish white, except apical portions of middle and hind tibiae and tarsi yellowish brown to darkish brown. Claws pectinate.

#### Description.

Female. Body length about 7.2 mm. Fore wing length about 5.8 mm. Ovipositor sheath length about 1.7 mm.

**Head.** Face (Fig. 12) convex centrally, approximately 1.5 times as wide as long, with leathery texture and fine punctures, distance between punctures 0.3 to 1.5 times diameter of puncture; upper margin with weak median elongate tubercle. Clypeus smooth, median portion transversely convex, with sparse, fine punctures; apical margin weakly convex forward, with light brown hairs. Mandible relatively short and wide, with sparse, fine punctures, upper tooth slightly wider and longer than lower tooth. Cheek and gena with fine granular texture. Malar space approximately 0.5 times as long as basal width of mandible. Gena almost impunctate, slightly convergent posteriorly, in lateral view about 0.6 times as long as width of eye. Vertex (Fig. 13) with texture as that of gena, lateral portion between posterior ocellus and eye with fine punctures. Postero-ocellar line about 0.9 times as long as ocular-ocellar line. Upper and lateral portions of frons approximately flat, with distinct, dense, fine punctures; lower portion concave centrally, slightly rough, with dense, indistinct, fine punctures. Antenna relatively thin and long, with 32 flagellomeres, each segment longer than wide; ratio of length of flagellomere 1:2:3:4:5 is 6.3:4.7:4.3:4.2:3.8. Occipital carina complete and strong, joining hypostomal carina above base of mandible.

**Mesosoma.** With fine leathery texture. Lateral concave part of pronotum with weak, short transverse wrinkles; upper-posterior portion with weak, fine punctures, distance between punctures 0.5 to 2.0 times diameter of puncture. Mesoscutum evenly convex, with dense punctures, distance between punctures 0.2 to 1.0 times diameter of puncture; notauli very weak, vestigial on front portion of mesoscutum. Scutellum slightly convex, with very sparse, fine punctures; without lateral carina except lateroanterior corner. Postscutellum transverse, anterior portion concave, with short longitudinal wrinkles. Mesopleuron (Fig. 14) with even, fine punctures, distance between punctures 0.2 to 1.0 times diameter of puncture. Upper end of epicnemial carina almost reaching mid-height of front margin of mesopleuron, distant from front margin of mesopleuron. Speculum present, small. Mesopleural fovea deep. Metapleuron with texture as that of mesopleuron, but slightly coarser. Anterior portion of submetapleural carina convex, as a broad lobe, translucent. Wings hyaline. Fore wing vein 1cu-a distal of 1/M, distance between them about 0.4 times length of 1cu-a. Areolet obliquely quadrate, receiving vein 2m-cu approximately at apical 0.25. 2-Cu slightly longer than 2cu-a. Hind wing vein 1/cu about 3.0 times as long as cu-a. Legs comparatively gracile. Claws pectinate. Ratio of length of hind tarsomere 1:2:3:4:5 is 1.0:4.6:3.3:1.8:2.2. Length of hind claw approximately 1.7 times as long as largest width of hind tarsomere 5 in lateral view. Propodeum (Fig. 15) rough, with irregular short wrinkles. Posterior transverse carina complete and strong. Pleural carina and median portion of lateral longitudinal carina (between spiracle and posterior transverse carina) present. Petiolar area with flabelliform wrinkles. First and second lateral areas with indistinct punctures. Propodeal spiracle small, round.

**Metasoma.** Terga with dense punctures, but slightly finer than on mesosoma, posterior margins smooth. First tergum approximately 1.6 times as long as apical width, evenly narrowed towards base, with fine transverse lines. Median dorsal carina indistinct. Anterior portion of dorsolateral carina, before spiracle, strong; posterior portion weak. Spiracle convex, located slightly before middle of tergum. Secong and following terga with leathery texture and fine transverse lines. Second tergum approximately 0.9 times as long as apical width. Thyridium oblique, basal margin reaching basal margin of second tergum. Third tergum approximately 0.75 times as long as apical width. Ovipositor sheath approximately 0.7 times as long as hind tibia, 1.3 times as long as first tergum. Ovipositor strongly compressed, weakly upcurved.

**Figures 11–15.**
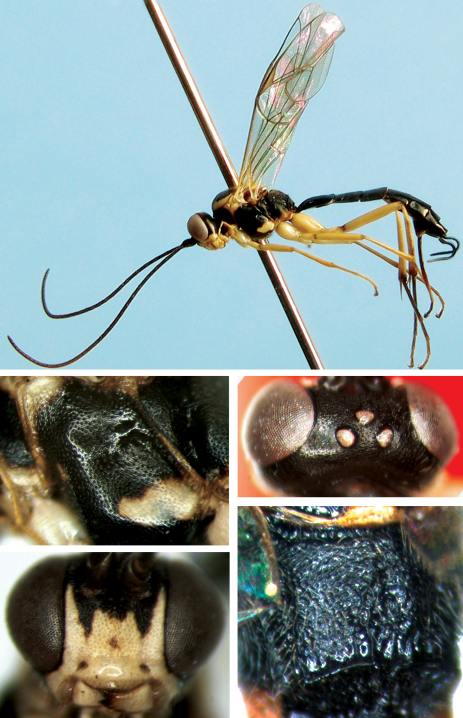
*Cryptopimpla flavipedalis* sp. n. Female **11** Body, lateral view **12** Face **13** Vertex **14** Mesopleuron **15** Propodeum.

**Color.** (Fig. 11). Black, except the following. Apical portion of flagella slightly blackish brown. Ventral side of scape, apical margin of pedicel, face (except upper margin irregularly black), clypeus, mandible except teeth, cheek, maxillary and labial palpi (except apical segments slightly filemot), lower portion of anterior margin, posterior margin and upper-posterior corner of pronotum, lateral margin widely and two narrow longitudinal median strips of mesoscutum, tegula, scutellum, posterior margin of postscutellum, a small spot on lower-anterior portion and irregular transverse band on lower-posterior portion of mesopleuron, lower portion of metapleuron, front legs, coxae, trochanters and femora of middle and hind legs yellowish white. Apical portion of middle tibia and tarsomeres yellowish brown to dark brown. Ventral and dorsal sides of hind tibia yellow, lateral sides dark brown. Hind tarsomeres dark brown, apical portions of each tarsomere yellowish brown. Stigma yellow. Veins brownish black.

#### Remarks.

This new species is similar to *Cryptopimpla helvicoxis* Chandra & Gupta, 1977, but can be distinguished from the latter by the following combination of characters: gena almost impunctate, hind wing vein 1-cu about 3.0 times as long as cu-a, posterior transverse carina complete and strong, hind coxae and femora yellow. *Cryptopimpla helvicoxis*: gena with dense, distinct punctures, hind wing vein 1-cu about 2.0 times as long as cu-a, posterior transverse carina interrupted or weak on either side of the middle, hind coxae and femora black.

### Cryptopimpla maculifacialis

Sheng sp. n.

urn:lsid:zoobank.org:act:F8E59EAB-64AD-46A5-9B37-FB1C64B4CB87

http://species-id.net/wiki/Cryptopimpla_maculifacialis

Figs 16–19

#### Etymology.

The specific name is derived from the face, which has a dark fleck.

#### Material examined.

*Holotype*: female, CHINA: Shuangjiang Forest Farm, Ji’an, Jiangxi Province, 15 June 2008, leg. Yi Kuang. *Paratypes*: 1 male, CHINA: Matubei, 320m, Quannan, Jiangxi Province, 6 May 2009, leg. Shi-Chang Li. 1 male, CHINA: Wokou, 320m, Quannan, Jiangxi Province, 13 May 2009, leg. Shi-Chang Li. 1 male, CHINA: Citangbei, 378m, Quannan, Jiangxi Province, 27 May 2009, leg. Shi-Chang Li. 1 male, CHINA: Shuangjiang Forest Farm, Ji’an, Jiangxi Province, 1 June 2009, leg. Da-Lin Li.

#### Diagnosis.

Mesosoma with fine leathery texture. Ovipositor sheath shorter than first tergum. Face white with median longitudinal black band. Flagellomeres 10 to 16 white. Scutellum yellowish white. Basal-median portion of first tergum white to yellowish white.

#### Description.

Female. Body length about 8.3 mm. Fore wing length about 6.2 mm. Ovipositor sheath length about 1.6 mm.

**Head.** Face (Fig. 17) slightly convergent ventrally, approximately 1.7 times as wide as long, median portion evenly convex, with dense punctures, distance between punctures 0.1 to 0.3 times diameter of puncture; lateral portion with punctures almost contacting each other as transverse wrinkles, upper margin with small median tubercle. Clypeus approximately 2.2 times as wide as long, smooth, evenly convex, basal portion with sparse, fine punctures, apical portion almost impunctate; apical margin thick, convex, with relatively long hairs. Mandible strong, with weak, fine punctures, distance between punctures 0.5 to 2.0 times diameter of puncture. Upper tooth sharp, longer than lower tooth. Malar space, gena, vertex and frons with fine leathery texture. Malar space approximately 0.5 times as long as basal width of mandible, with indistinct punctures. Gena slightly convergent posteriorly, with distinct punctures, distance between punctures 0.2 to 1.5 times diameter of puncture. Vertex (Fig. 18) with punctures slightly denser than on gena. Interocellar area slightly convex. Postocellar line approximaly 1.1 times as long as ocular-ocellar line. Frons almost flat, with dense punctures, distance between punctures 0.1 to 0.5 times diameter of puncture, almost contacting each other transversely. Upper median portion, below median ocellus, concave. Antenna with 34 flagellomeres, each flagellomere longer than wide; ratio of length from first to fifth flagellomeres: 6.4:4.4:4.2:4.0:3.7. Occipital carina complete, lower end joining hypostomal carina above base of mandible.

**Mesosoma.** Mesosoma with fine leathery texture. Anterior margin of pronotum with elongate punctures, lateral concavity with short transverse wrinkles, posterior portion with dense punctures, distance between punctures 0.2 to 0.5 times diameter of puncture. Mesoscutum evenly convex, with punctures as on posterior portion of pronotum. Without notaulus. Scutellum slightly convex, with sparse, shallow, irregular punctures, distance between punctures 0.2 to 3.0 times diameter of puncture, without lateral carina. Postscutellum transverse, with weak punctures, anterior portion with transverse concavity and deep lateral pit. Mesopleuron (Fig. 19) with dense punctures, distance between punctures 0.2 to 1.0 times diameter of puncture. Upper end of epicnemial carina reaching level of lower third of front margin of mesopleuron, distant from front margin of mesopleuron. Mesopleural fovea deep. Without speculum. Metapleuron with denser, finer punctures than mesopleuron. Front portion of submetapleural carina convex, as a broad lobe. Without juxtacoxal carina. Wings brownish hyaline. Vein 1cu-a distal of 1/M, distance between them approximately 0.4 times length of 1cu-a. Areolet quadrate, vein 3rs-m distinctly longer than 2rs-m, receiving vein 2m-cu approximately at 0.7 distance from vein 2rs-m to 3rs-m. Vein 2-Cu slightly longer than 2cu-a. Hind wing vein 1/cu about 2 times as long as cu-a. Legs comparatively robust. Ratio of length of hind tarsomeres 10.0:4.6:3.2:1.7:2.1. Claws simple. Propodeum evenly convex, with dense, fine, indistinct punctures. Posterior transverse carina complete and strong. Pleural carina weak. Propodeal spiracle almost round.

**Metasoma.** First tergum approximately 1.6 times as long as apical width, evenly narrowed towards base, rough, with dense, indistinct punctures, lateral portion with short longitudinal wrinkles, median dorsal carina indistinct, basal portion of dorsolateral carina present, spiracle small, weakly convex, slightly before middle of tergum. Second tergum about 0.9 times as long as apical width, with dense punctures, distance between punctures 0.1 to 0.5 times diameter of puncture, apical margin narrowly smooth, basal margin with transverse thyridium, spiracle distinctly convex. Third tergum with finer punctures than second tergum. Fourth tergum almost smooth, indistinctly punctate. Following terga smooth, with more or less clear fine transverse lines. Ovipositor sheath approximately 0.5 times as long as hind tibia, 0.9 times as long as first tergum. Ovipositor evenly upcurved and compressed.

**Figures 16–19.**
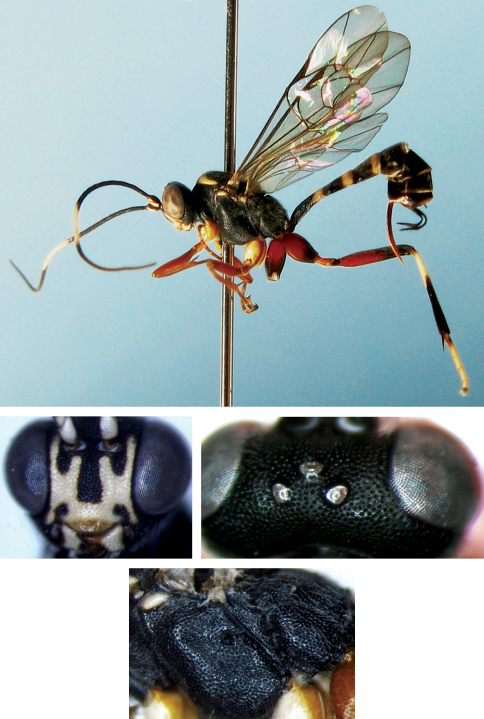
*Cryptopimpla maculifacialis* sp. n. Female **16** Body, lateral view **17** Face **18** Vertex **19** Mesopleuron.

**Color.** (Fig. 16). Black, except the following. White portions: ventral profile of scape and pedicel, flagellomeres 10 to 16 and apical portion of 9, face except upper narrow margin and median longitudinal band and short elongate spot below antennal socket, clypeus except apical half yellowish brown, mandible except teeth, maxillary palpus except apical 2 segments yellowish brown, labial palpus except apical segment yellowish brown, upper-posterior corner of pronotum, elongate spot on latero-anterior portion of mesoscutum, subalar ridge, scutellum, ventral profile of front coxa and trochanter, ventral profile of middle coxa, basal-median portion of tergum 1, apical bands of terga 1 to 3, narrow apical margins of terga 4 to 7. Legs reddish brown. Basal-ventral sides of front and middle femora, dorsal sides of basal portions of front and middle tibiae, basal portion of hind tibia and tarsomeres 1 to 4 white. Apex of hind coxa, hind trochantellus, apical portion of hind femur, apical half of hind tibia, and hind tarsomere 5 black. Stigma dark brown. Veins brownish black.

**Male.** Body length 8.5 to 8.8 mm. Fore wing length 6.3 to 6.5 mm. Antenna with 33 to 34 flagellomeres. Face yellow with irregular transverse black band to almost entirely black.

#### Variation.

The face of one male specimen is almost enitrely black, with only very narrow sublateral yellow lines. Tergum 1 of male with basal and apical portions white to unevenly brownish yellow with two small longitudinal black spots.

#### Remarks.

Similar to *Cryptopimpla flavifacialis* Sheng, but can be distinguished from the latter in having the first tergum approximately 1.6 times as long as apical width, mesosoma with fine leathery texture, metapleuron with denser and finer punctures than mesopleuron, face white with median longitudinal black band, flagellomeres 10 to 16 white, scutellum yellowish white, basal median portion of tergum 1 white to yellowish white. *Cryptopimpla flavifacialis*: first tergum 1.8 to 1.9 times as long as apical width, mesosoma with smooth texture, metapleuron with punctures as on mesopleuron, face yellow, without median longitudinal black band, median to subapical portion of flagella yellowish brown, without white portion, scutellum black, basal portion of tergum 1 black.

### Cryptopimpla rufipedalis

Sheng sp. n.

urn:lsid:zoobank.org:act:1D3E113C-E13F-46E6-8AFE-DD5712063ABC

http://species-id.net/wiki/Cryptopimpla_rufipedalis

Figs 20–24

Cryptopimpla helvetica Brauns 1901: Sheng. Acta Zootaxomica Sinica, 2005, 30:417.

#### Etymology.

The name of the new species is based on the leg being brownish red.

#### Material examined.

*Holotype*: female, CHINA: Baihe, Jilin Province, 14 July 2002, leg. D-J Hao.

#### Diagnosis.

Hind wing 1/cu approximately 4.7 times as long as cu-a. Posterior transverse carina of propodeum weakly present centrally. Face black, median portion of inner orbit yellow; legs almost entirely brown. Apical portion of tergum 1, terga 2 to 3, basal portion of tergum 4 and subposterior margin of tergum 5 reddish brown. Stigma yellowish brown.

#### Description.

Female. Body length about 7.0 mm. Fore wing length about 5.8 mm. Ovipositor sheath length about 1.5 mm.

**Head.** Head with fine leathery texture. Inner orbits parallel. Face (Fig. 21) approximately 1.45 times as wide as long. Median portion slightly convex, with weak, fine punctures, distance between punctures 0.2 to 1.0 times diameter of puncture. Upper margin with median concavity, without tubercle. Clypeus convex, 2.4 times as wide as long, basal portion with fine, indistinct punctures, apically almost smooth, apical margin thick, evenly convex. Mandible strong, with weak, fine punctures, upper tooth longer than lower tooth. Malar space approximately 0.6 times as long as basal width of mandible, with fine punctures. Gena slightly convergent backwardly, with distinct punctures, distance between punctures 0.2 to 1.0 times diameter of puncture on lower portion, 0.2 to 3.0 times on upper portion. Vertex (Fig. 22) almost smooth, with distinct punctures, distance between punctures 0.2 to 1.5 times diameter of puncture. Interocellar area slightly convex. Postocellar line 0.8 times as long as ocular-ocellar line. Frons almost flat, with texture as that of face. Antenna with 34 flagellomeres, each of them longer than wide, ratio of length from first to fifth flagellomeres: 1.5:1.2:1.0:0.9:0.8. Occipital carina complete.

**Mesosoma.** With fine, unclear leathery texture. Pronotum with distinct punctures, distance between punctures 0.2 to 0.5 times diameter of puncture, upper half of lateral concavity with indistinct, short transverse wrinkles. Mesoscutum correspondingly convex, with punctures as on pronotum, but median portion of median lobe comparatively finely and indistinctly punctate. Without notaulus. Scutellum evidently convex, with punctures as on lateral lobe of mesoscutum. Postscutellum transverse, with indistinct punctures, latero-anterior portion with small deep fovea. Mesopleuron (Fig. 23) with dense punctures, distance between punctures 0.1 to 1.5 times diameter of puncture. Upper end of epicnemial carina reaching level of lower third of front margin of mesopleuron, distant from front margin. Without speculum. Metapleuron with finer and denser punctures than mesopleuron, distance between punctures 0.1 to 0.5 times diameter of puncture. Without Juxtacoxal carina. Submetapleural carina complete, anterior portion strongly convex as a broad lobe. Wings gray-brownish hyaline. Vein 1cu-a distal of 1/M, distance between them 0.3 times 1cu-a. Areolet slanting quadrate. Vein 3rs-m distinctly longer than 2rs-m. 2m-cu slightly basal of lower-outer corner of areolet. Vein 2-Cu slightly longer than 2cu-a. Hind wing vein 1/cu about 4.7 times as long as cu-a. Legs comparatively long. Ratio of length of hind tarsomeres10.0:4.0:3.0:2.0:2.5. Claws simple. Propodeum (Fig. 24) evenly convex, with punctures as on metapleuron, pleural and median portion of posterior transverse carinae present. Propodeal spiracle almost round.

**Metasoma.** Terga with fine leathery texture. First tergum approximately 1.8 times as long as apical width, evenly narrowed toward base, convex longitudinally, anterior portion of postpetiole, near spiracle, with dense punctures, posterior portion sparsely punctate, median portion of posterior margin smooth. Without median dorsal carina. Basal and apical portions of dorsolateral carina present. Spiracle small, round, convex, located at basal 0.35 of first tergum. Second tergum approximately as long as apical width, with fine, irregular, indistinct punctures, apical margin almost smooth, thyridium indistinct. Third tergum approximately 0.9 times as long as apical width, lateral margins almost parallel, with texture as second tergum but punctures comparatively finer, sparser than second tergum. Ovipositor sheath approximately 0.6 times as long as hind tibia, as long as first tergum. Ovipositor evenly upcurved and compressed.

**Figures 20–24.**
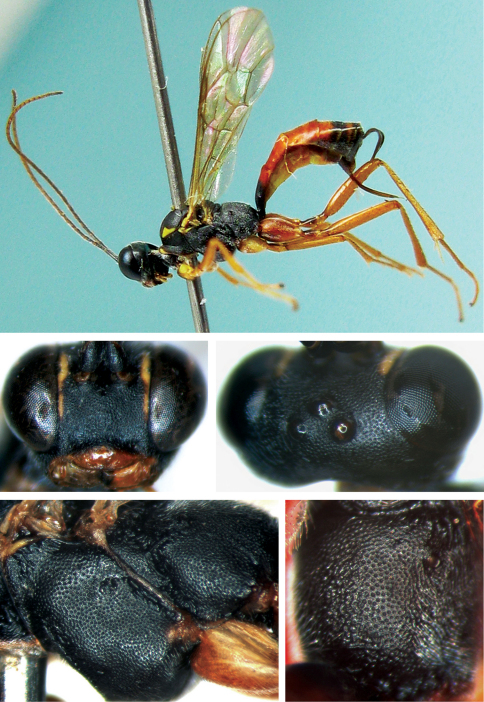
*Cryptopimpla rufipedalis* sp. n. Holotype. Female **20** Body, lateral view **21** Face **22** Vertex **23** Mesopleuron **24** Propodeum.

**Color.** (Fig. 20). Black, except the following. Apical portion of pedicel slightly taupe. Apical portion of flagella brownish black. Apical half of clypeus and base of mandible reddish brown. Median portion of inner orbit, anterior margin and upper-posterior corner of pronotum, triangular spot on latero-anterior margin of mesoscutum, tegula, transverse spot on subalar ridge, dorsal profiles of front and middle coxae yellow. Maxillary and labial palpi dust-coloured. Legs reddish brown. Basal portions of ventral profiles of coxae and dorsal profiles of trochanters more or less black. Apical portion of tergum 1, terga 2 and 3, basal portion of tergum 4 and subposterior margin of tergum 5 reddish brown. Stigma yellow. Veins dark brown.

#### Remarks.

This new species is similar to *Cryptopimpla helvetica* but can be distinguished from the latter by the following combination of characters. Ovipositor sheath about 0.6 times as long as the length of hind tibia. Ovipositor curved upwardly. Second tergum with dense and distinct punctures. 1/cu straight, approximately 4.7 times as long as cu-a. Median portion of posterior transverse carina of propodeum weakly present. Face black, inner orbits yellow. Ventral sides of fore and mid coxae reddish brown, dorsal sides yellow. Anterior margin of pronotum and latero-anterior portion of mesonotum yellow. *Cryptopimpla helvetica*: ovipositor sheath approximately 0.9 times as long as the length of hind tibia. Ovipositor straight. Second tergum with sparse and indistinct punctures. 1/cu bowing inward, approximately 3.5 times as long as cu-a. Posterior transverse carina of propodeum complete and strong. Inner orbits and face entirely black. Fore and mid coxae black or brownish black. Pronotum and mesonotum entirely black.

### Cryptopimpla brevis

Sheng, 2005

Cryptopimpla brevis Sheng 2005. Acta Zootaxomica Sinica, 30: 415.

#### Specimens examined.

1 female, CHINA: Huhehaote, Inner Mongolia, 29 August 1995, Mao-Ling Sheng.

### Cryptopimpla henanensis

Sheng, 2005

Cryptopimpla henanensis Sheng 2005. Acta Zootaxomica Sinica, 30:416.

#### Specimens examined.

1 female, CHINA: Laojieling Natural Reserve, 1350 m, Henan Province, 17 July 1998, Mao-Ling Sheng. 3 females, CHINA: Baiyuanshan Natural Reserve, 1400m, Henan Province, 24 to 25 July 2003, Ji-Xing Fan, Wen-Jun Wang. 2 females 5 males, CHINA: Liupanshan, 1820m, Ningxia Hui Autonomous Region, 4 to 18 August 2005, Mao-Ling Sheng. 1 female, CHINA: Taibai Mt. 1600–1800m, Shanxi Province, 7 July 2007, Xi Zhu. 1 female, CHINA: Beishan Forest Farm, Huzhu, Qinghai Province, 2366m, 20 July 2010, Mao-Ling Sheng.

### Cryptopimpla taiwanensis

(Momoi, 1968)

Fintona taiwanensis Momoi 1968. Kontyu, 36(2):187.

Specimens not examined.

## Supplementary Material

XML Treatment for Cryptopimpla

XML Treatment for Cryptopimpla carinifacialis

XML Treatment for Cryptopimpla flavifacialis

XML Treatment for Cryptopimpla flavipedalis

XML Treatment for Cryptopimpla maculifacialis

XML Treatment for Cryptopimpla rufipedalis

XML Treatment for Cryptopimpla brevis

XML Treatment for Cryptopimpla henanensis

XML Treatment for Cryptopimpla taiwanensis
